# The Effects of Modified Game Schedules on Injury Rates in the National Hockey League (NHL)

**DOI:** 10.7759/cureus.46898

**Published:** 2023-10-12

**Authors:** Quinn T Ehlen, Joseph P Costello, Jaxon D Wagner, Jacob L Cohen, Lauren C Baker, Michael G Rizzo, Lee D Kaplan

**Affiliations:** 1 Orthopedics, University of Miami Miller School of Medicine, Miami, USA; 2 Orthopedics, University of Miami Miller School of Medicine, Jackson Memorial Hospital, Miami, USA; 3 Orthopedic Surgery, University of Miami Miller School of Medicine, Jackson Memorial Hospital, Miami, USA; 4 Sports Medicine, University of Miami, Miami, USA

**Keywords:** ice hockey, sports medicine, covid-19, injury rates, epidemiology

## Abstract

Background

Due to the COVID-19 pandemic, many professional sports leagues such as the National Hockey League (NHL) made significant changes to their schedules and operating procedures. Changes included a modified 2019-2020 playoff format, the removal of the 2020-2021 preseason, and condensed game schedules. Though these modifications were made in an effort to protect players from COVID-19, they resulted in decreased training time and preparation. The purpose of this study was to assess the impact of these changes on the rate of player injuries in the NHL both after the resumption of the midseason stoppage and during the subsequent seasons.

Hypothesis/purpose

Changes to the NHL schedule amid the COVID-19 pandemic resulted in a significant increase in player injury rates.

Methods

NHL injuries were obtained from an NHL injury database for the 2018-2019 through the 2021-2022 seasons. The date of injury, date of return, injury description, player age, and player position were recorded. Injury rates were calculated as the number of total athlete injuries per 1000 game exposures (GEs). The primary outcome was the injury proportion ratio (IPR) when comparing the injury rates of the post-COVID-19 season with baseline seasons. Secondary measures analyzed injuries based on age, anatomic location, month in the season, position, length of injury, season-ending injuries, and recurring injuries.

Results

A total of 4604 injuries were recorded between 2018 and 2022. The modified 2019-2020 playoffs had significantly higher rates of injury (IPR = 1.84, 95% confidence interval {CI} = 1.36-2.49) with more game exposures per week. The 2020-2021 season had significantly higher rates of overall player injury compared to baseline seasons (IPR = 1.19, 95% CI = 1.09-1.30) and also had a higher rate of season-ending injuries (IPR = 1.71, 95% CI = 1.38-2.11). Most injuries occurred in the first few months of the 2020-2021 season. There was no significant difference in injury rate based on age group and no significant difference in the average length of injury between seasons.

Conclusion

Increases in injury rates could be due to decreased offseason training between seasons, the elimination of preseason games, and increased game density. Decreasing typical training timelines and eliminating the preseason to rapidly return to normal competition after unexpected events (pandemics, lockdowns, etc.) may pose a risk to player safety in the NHL. These findings should be considered before future schedule changes in professional hockey.

## Introduction

The 2020 COVID-19 pandemic halted the 2019-2020 National Hockey League (NHL) midseason, a month before playoffs were scheduled to begin. It was uncertain how the NHL would finish the season. Due to lockdowns and other restrictions implemented, players were absent from the team for four months before returning in July for a two-week training camp, consistent with standard preseason training camps [1]. The season resumed on August 1, 2020, in a modified format [2]. The modified COVID-19 playoff consisted of a brief 32-game qualifying round (16 teams played four games each) before the playoffs began on August 11 and concluded on September 28 [2]. While the NHL had zero confirmed COVID-19 cases among players during their modified season [2], concern existed for increased injury rates due to schedule and operational changes.

As a high-velocity contact sport, injuries are prevalent in professional hockey, with 51% of NHL players missing at least one game due to injury every season [3]. Some of these injuries are fatigue-related, and it has been suggested that decreased recovery time between games results in higher injury rates for NHL players [4]. It is also known that less recovery can manifest as overtraining syndrome, which can lead to decreased performance and increased injury likelihood [5].

Playoff hockey specifically has been demonstrated to result in more physical play than the regular season [6]. This style of rougher play manifests as a higher incidence of body checks in the playoffs, the most common mechanism of injury for NHL players [7]. Because muscle fatigue can impair postural stability and extremity control, there is potential for increased injury rates when fatigued players are competing in a rougher style of play [8,9]. Returning to playoff-intensity hockey with a condensed schedule but without adequate time for training and conditioning could contribute to higher injuries during the 2019-2020 playoffs.

Furthermore, following the 2019-2020 season, which was interrupted by COVID-19, many ice arenas and training facilities were still closed due to COVID-19 outbreaks [10]. Despite the diminished training, the NHL opted to forgo the preseason for the 2020-2021 season, with the season beginning on January 13, 2021. Historically, the preseason starts in September with the regular season beginning in October. The 2020-2021 season was delayed a few months because of the four-month COVID-19 break during the modified 2019-2020 season, which delayed the 2019-2020 playoffs.

The return to play, with diminished offseason training and without the typical preseason, has led to the question of how these changes impacted player safety. While there has been literature published on injuries in the NHL, most commonly regarding mechanisms of injury, studies have not analyzed the possible effects of COVID-19-related changes on the incidence of player injury [11-13].

Due to the lack of previous analysis, the current study sought to evaluate injury rates during and after the 2019-2020 NHL season. We hypothesize that the modified 2019-2020 NHL playoffs had significantly higher rates of injuries compared to other NHL playoffs. Also, we hypothesize that the 2020-2021 season will have significantly higher rates of injury compared to pre-pandemic, standard NHL seasons. To our knowledge, this study is the first report of the COVID-19 impact on the epidemiology of NHL injuries. Findings from this study can be applied to future scheduling and training for the NHL and other ice hockey leagues.

## Materials and methods

Data collection

The study was considered exempt by our institutional review board, and informed consent was not sought as all data was publicly available. All injuries from the 2018-2022 NHL seasons were obtained through a review of injury transactions, injury reports, and injured reserve (IR) placements. Data was sourced from NHL injury databases, media reports, and other news sources (ProSportsTransactions.com, Hockey-Reference.com, ESPN.com, and CBSSports.com). These databases have been cited for several professional sports epidemiological studies [14-16]. Each recorded injury was verified using historical reports from NHL.com and Hockey-Reference.com. Information from Hockey-Reference.com is provided by Sportradar US, which is the official statistics partner of the NHL. All NHL players who suffered injuries while in the NHL during the 2018-2022 seasons were included in this study. Players for minor league affiliates were not included.

Definitions

Season

An NHL season was considered to be the first preseason game of the year until the playoffs were completed. The seasons are identified by the year in which it began. For example, the 2021-2022 season is considered the 2021 season.

Injury

An injury was considered as any physical ailment that caused a player to miss a game. Illnesses, personal absences, and offseason injuries were excluded from the analysis.

Injury Length

The injury length was calculated as the total time elapsed from the date of injury to the date of their first game back competing.

Season-Ending Injury

An injury was considered season-ending if the player did not return to play in a game during that same season.

Multiple Injuries

If the same player was injured more than one time in a season regardless of anatomic location, the player was considered to have multiple injuries. For example, if a player injured their knee, returned to play, and then injured their shoulder, this would be a multiple injury. If a player injured their knee, returned to play, and then injured their knee again, this would also be considered a multiple injury.

Anatomic Location

Injuries were categorized based on anatomic locations as reported in the databases. For injury descriptions that were vague, subsequent reports were found to correctly categorize the injury location.

Age

The age of the player was determined by taking their date of injury less their date of birth, found on Hockey-Reference.com.

Position

Player position was found on Hockey-Reference.com, including forward, defense, and goalie.

Game Exposure (GE)

A game exposure is defined as a single player participating in an individual game, regardless of their total time on the ice in that game. This does not include practices or training outside of games. Total seasonal game exposures and playoff game exposures were calculated by summing all the game exposures throughout the season and playoff, respectively. Rates of injury were calculated as injuries per 1000 GEs.

Game Density

Game density was calculated by taking the number of games divided by the number of days in which those games occurred. Higher game density indicates more game exposures in fewer days.

Statistical analysis

All statistical analysis was done using Statistical Package for Social Sciences (SPSS) version 28 (IBM SPSS Statistics, Armonk, NY). Preseason, regular season, playoff, and overall season injury rates were calculated for the 2018-2019 through the 2021-2022 seasons. The NHL eliminated the preseason for the 2020-2021 season, so there were no preseason injuries recorded for that year. The baseline seasons (2018, 2019, and 2021) were used to represent the standard NHL seasons, without significant schedule or protocol changes. Injury incidence was calculated as total injuries per 1000 GEs. The injury proportion ratio (IPR) is defined as the ratio of injury incidence between seasons. An IPR > 1 indicates higher injury rates in the studied season. An IPR is statistically significant if the 95% confidence interval (CI) does not contain 1.0, indicating p < 0.05. Relevant frequency statistics including the anatomic location of the injury, player position, age, and length of injury were extracted, and further exploration was conducted by stratifying with these variables.

## Results

Overall injuries

From 2018 to 2022, NHL players sustained 3215 injuries causing them to miss games. During this time, there were 334386 total GEs, resulting in a cumulative incidence of 9.61 injuries per 1000 GEs (Table 1).

**Table 1:** NHL injuries by position, age, and average length of injury by season (n {%}) NHL: National Hockey League

Season	2018-2019	2019-2020	2020-2021	2021-2022
Total injuries	769	761	683	1002
Game exposures	86043	97049	61778	89516
Forwards (%)	273 (56.6)	259 (59.3)	218 (57.0)	337 (56.7)
Defensemen (%)	435 (35.5)	451 (34.0)	389 (31.9)	568 (33.6)
Goalies (%)	61 (7.9)	51 (6.7)	76 (11.1)	97 (9.7)
>40 years old	3 (0.4)	2 (0.3)	4 (0.6)	5 (0.5)
30-39 years old	233 (30.3)	206 (27.1)	215 (31.5)	296 (29.5)
20-29 years old	520 (67.6)	534 (70.2)	455 (66.6)	698 (69.7)
<20 years old	13 (1.7)	19 (2.5)	9 (1.3)	3 (0.3)
Mean age	28 ± 4.4	28 ± 4.3	28 ± 4.1	28 ± 4.3
Average length of injury	36 ± 68	51 ± 84	41 ± 68	22 ± 28

Player characteristics

**Figure 1:**
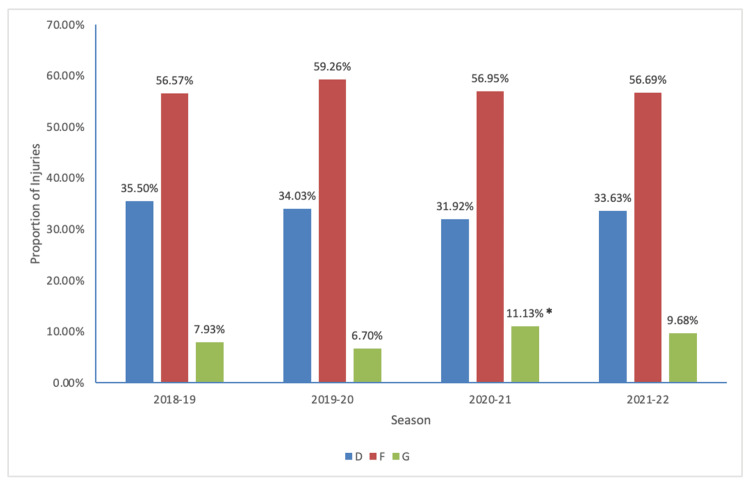
Proportion of injuries by player position *Significant increase in goalie injuries relative to pre-pandemic levels (p = 0.01) F, forwards; D, defensemen; G, goalies

Forwards had the highest proportion of injuries in each season even after controlling for the number of each position on the ice at a given time (Table 1). During the 2020-2021 season, goalies had a significantly higher rate of injury relative to other years (p < 0.05) (Figure 1). There was no significant difference in the average age of injury between seasons. When stratifying the injuries into respective age categories, there was no significant difference between older or younger age brackets across seasons (Table 1).

2019-2020 playoff injuries

**Figure 2:**
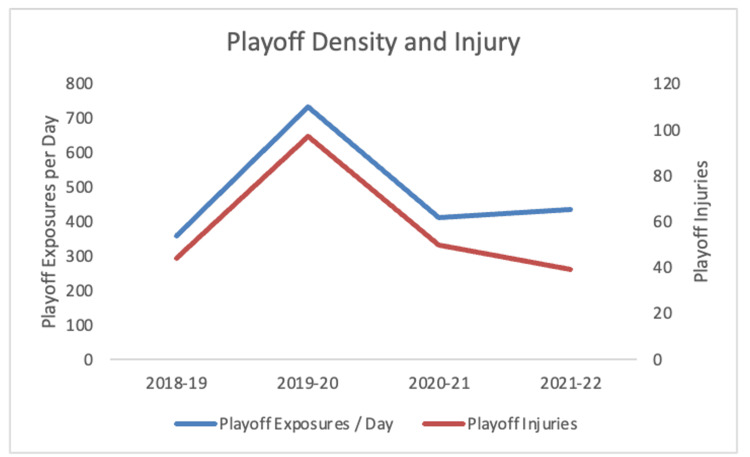
Relationship between playoff game density and the number of playoff injuries in the studied seasons (r2 = 0.92, p = 0.041)

During the 2019-2020 modified playoff schedule, there was a significantly higher rate of injury with IPR = 1.84 (95% CI = 1.36-2.49), compared to the average of baseline years (Table 2). The playoff game density, represented as playoff exposures per day, was significantly higher in 2019-2020 relative to the pre-pandemic density (Table 3). Over the analyzed seasons, playoff game density and playoff injuries were significantly correlated with r2 = 0.92 (p = 0.04) (Figure 2).

**Table 2:** NHL total season injuries, playoff injuries, season-ending injuries, and multiple injuries per 1000 GEs with respective IPRs For playoff values, the 2019-2020 season is the affected year. For regular season values, the 2020-2021 season is the affected year. (a) Statistically significant increase in injury incidence for 2020-2021 (IPR = 1.19, 95% CI = 1.09-1.30), (b) statistically significant increase in playoff injury incidence for 2019-2020 (IPR = 1.84, 95% CI = 1.36-2.49), and (c) statistically significant increase in season-ending injury incidence for 2020-2021 (IPR = 1.71, 95% CI = 1.38-2.11) *P < 0.0001 IPR, injury proportion ratio; NHL, National Hockey League; GEs, game exposures; CI, confidence interval

	Average baseline incidence	COVID-19-affected year	IPR
Total injuries (a)	9.29	11.06	1.19*
Playoff injuries (b)	1.24	2.28	1.84*
Season-ending injuries (c)	1.17	2.01	1.71*
Multiple injuries	3.54	3.51	0.99

**Table 3:** Density of game schedules and average rest time between games for each season *Significant increase relative to pre-pandemic levels (p < 0.01)

Season	2018-2019	2019-2020	2020-2021	2021-2022
Exposures/day	330.93	399.38*	367.73*	275.43
Games/day	0.34	0.31	0.33	0.27
Average days between games	2.92	3.18	3.00	3.65
Playoff exposures/day	358.51	732.83*	412.26*	436.58

2020-2021 season injuries

**Figure 3:**
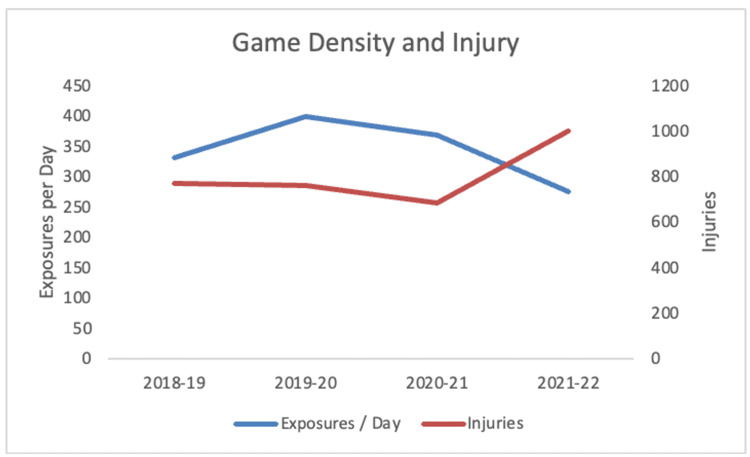
Relationship between game density and the number of injuries in the studied seasons (r2 = 0.70, p = 0.16)

**Figure 4:**
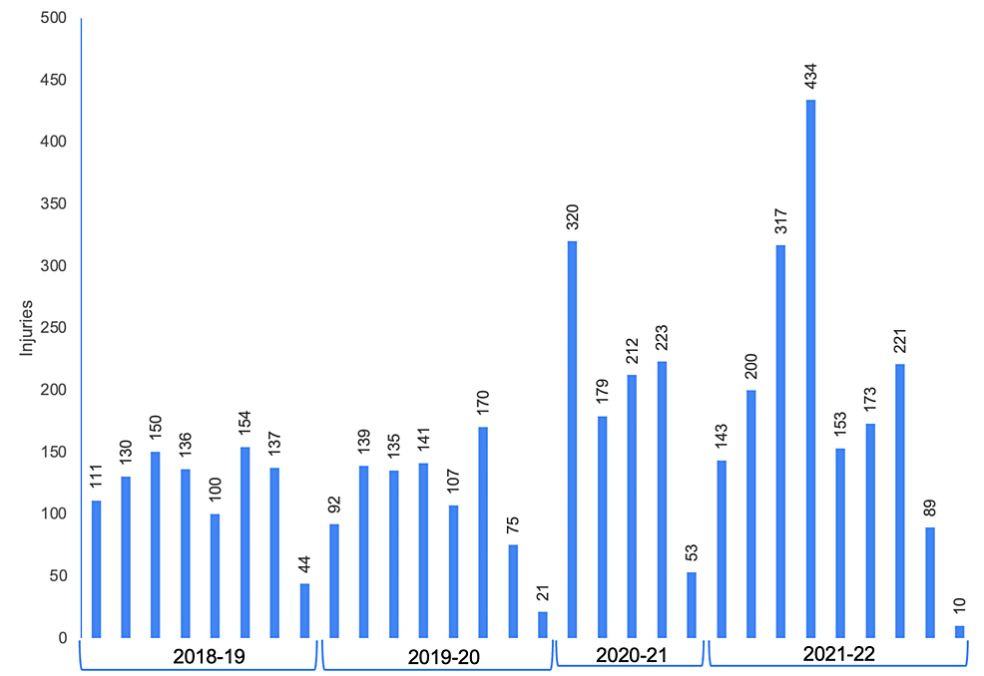
Total injuries in each season separated by month

During the 2020-2021 season, the rate of injury was significantly higher compared to the average of baseline years with IPR = 1.19 (95% CI = 1.09-1.30) (Table 2). The 2020-2021 season had a significantly higher average number of athletic exposures (AEs) per day than the pre-pandemic 2018-2019 season (Table 3). The relationship between game density and the injuries for each season is depicted in Figure 3. The distribution of injuries during the months in which they occurred for each season is represented in Figure 4. The distribution of injuries by month for the 2020-2021 season has a positive skew (Figure 4).

Season-ending injuries

A total of 444 season-ending injuries were identified with 85, 94, 124, and 141 occurring in the 2018, 2019, 2020, and 2021 seasons, respectively. The rate of season-ending injuries was higher in 2020-2021 compared to the average of baseline years with IPR = 1.71 (95% CI = 1.38-2.11) (Table 2).

Multiple injuries

Rates of multiple injuries in the same season were not significantly different among any of the studied seasons (Table 2).

Injury by anatomic location

**Figure 5:**
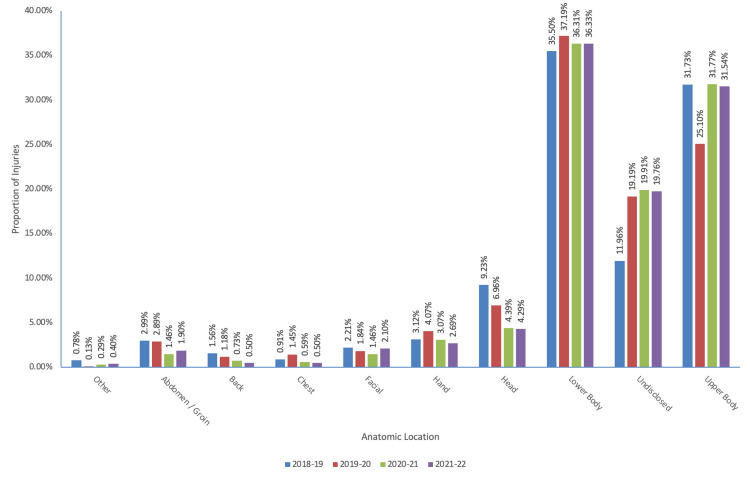
Proportion of NHL injuries based on anatomic locations during the respective seasons NHL: National Hockey League

The most frequent injuries reported were injuries of the lower body with 1168 reported over the four studied seasons. The 2020-2021 season had a significantly different distribution of injuries relative to the baseline seasons (Table 4). Notably, there was a higher incidence of hand, lower body, undisclosed, and upper body injuries relative to baseline. The respective IPRs can be found in Table 4. The incidence of head injuries decreased over the study years with an average of 1.74% per year (r2 = 0.91) (Figure 5).

**Table 4:** Incidence of NHL injuries based on anatomic locations during the respective seasons (a) 95% CI = 1.11-1.15, (b) 95% CI = 1.18-1.20, (c) 95% CI = 1.37-1.39, and (d) 95% CI = 1.25-1.27 *Significant increase in IPR for respective anatomic locations in the 2020-2021 year relative to average baseline incidence IPR, injury proportion ratio; NHL, National Hockey League; CI, confidence interval

Location	Average baseline incidence	2020-2021 incidence	IPR
Others	0.04	0.03	0.78
Abdomen/groin	0.24	0.16	0.69
Back	0.10	0.08	0.84
Chest	0.08	0.06	0.78
Facial	0.19	0.16	0.84
Hand (a)	0.30	0.34	1.13*
Head	0.62	0.49	0.79
Lower body (b)	3.39	4.01	1.19*
Undisclosed (c)	1.60	2.20	1.38*
Upper body (d)	2.78	3.51	1.26*
Total	9.29	11.06	1.19*

## Discussion

The COVID-19 bubble (2019-2020 playoffs)

After the halt of the 2019-2020 season, the NHL restarted the season in the playoffs. The modified playoff schedule included an increase in the number of games and teams involved, though without a similar increase in the length of time. While it is known that the playoffs generally see proportionately more injuries than the regular season, the injury rate for the 2019-2020 playoffs was significantly higher than baseline playoff rates, suggesting the possibility of an inciting factor [6]. The 2019-2020 playoffs had an extraordinarily high number of exposures per week with less recovery time between games. Decreases in game spacing have previously been proven to increase the risk of injury in the NHL [17]. Additionally, because we found a significant correlation between playoff density and injury, it is likely that the condensed schedule played a part in the increased injury rate during the 2019-20 playoffs.

Additionally, because of the extended four-month break between the regular season and the playoffs with limited training, the players were likely deconditioned with respect to hockey upon entering the playoffs. This deconditioning prior to the playoffs may have placed them at a higher risk of injury. Research also shows that players demonstrate improved on-ice performance with greater off-ice physical fitness [18,19]. As many players were limited in training capacity due to the closure of the training facilities, it is likely that their off-ice physical fitness suffered. Teams also typically monitor in-season changes for individual players, which allows trainers to create optimized training schedules that prevent detraining and injuries [20]. With players not at their team facilities, this kind of monitoring was limited.

No literature currently exists discussing the effects of COVID-19 on injury rates in the NHL; however, our results are supported by similar findings in other professional sports leagues following schedule and training changes as a result of COVID-19. There were increased injury rates in both the National Football League (NFL) [21-23] and the German Bundesliga soccer league [24] following the resumption of play after COVID-19 stoppages. It has also been shown more generally that return to play following a forced hiatus or disruption to routine training schedule may lead to an increased incidence of injuries, such as following the 2011 NFL lockout. Upon return, a significantly increased rate of Achilles tendon injuries was observed [25].

Injuries during the 2020-2021 season

Before the 2020-2021 season, the NHL decided to forgo the preseason. The general goal of the preseason is to allow players to become gradually more conditioned before transitioning to the rapid pace of play and the intense physicality of the regular season. Besides the elimination of the preseason itself, there were multiple other factors contributing to the decrease in the typical training time. One such circumstance was that numerous players had to sit out of training camp due to COVID-19 protocols [26]. There were also several ice rinks and training facilities closed due to virus outbreaks [10]. Overall, there was less time for players to return to peak physical form before high-level competition began. This likely contributed to the increase in injuries per exposure for the 2020-2021 season and the increase in season-ending injuries per exposure. Further, there were more injuries that occurred during the beginning months of the 2020-2021 season, alluding to possible deconditioning injuries.

Without proper preseason training, the players were at higher risk for the occurrence and severity of injuries. Under-training prior to competitive play has been reported to increase injury risk [27]. One study showed that NHL players who did not receive adequate training sessions in the offseason were more than three times more likely to have groin injuries or abdominal strains [28]. Although under-training is a risk, team training does have an optimal threshold before overtraining can begin to cause injuries as well. Other studies have noted decreases in injury occurrence and severity with preseason strength training and eccentric overload [29]. This theoretical ideal level maximizes team performance and minimizes injury risk [30].

Despite the increases in incidence and severity, there was no significant increase in the length of injury or multiple injuries per player during the 2020-2021 season. However, the season-ending injury rate did increase during the 2020-2021 season. As more players suffered season-ending injuries, there were more players who were not able to return to play and, therefore, were not able to get injured again. Regarding the lack of significant change in injury length despite the increase in season-ending injuries, because the length of injury was calculated for only players that returned to play that season, the length would likely not be affected by the increased season-ending injuries.

Examining the demographics of injuries, goalies specifically suffered higher injury rates for the 2020-2021 season compared to baseline. The position requires immense flexibility, and goalies historically have significantly more flexibility in the hip and groin musculature relative to forwards and defensemen [29]. With less preseason training, there is a higher risk of suffering lower body injuries, specifically to the groin [28]. Interestingly, age did not significantly affect the incidence of injury during the studied seasons.

The increase in injury rates and severity could also be due to the decreased recovery time between games. In the 2020-2021 season, there were 368 AEs per day, up from 331 in the pre-pandemic 2018-2019 season. This suggests that there were more games in fewer days throughout the season. A previous study indicated that an increased frequency of consecutive or “back-to-back” games can lead to more player fatigue and subsequent injuries [4]. Once again, the condensed schedule and urgency to squeeze in games following the COVID-19 break may have played a part in higher total and season-ending injury rates during the 2020-2021 season.

Overall injury trends

When analyzing the data over a longitudinal period, it seems that there may be an increasing injury rate overall. Future seasons will need to be taken into account to make such claims. It is known that technological advancements, such as lighter skates and equipment, continue to increase the speed of play [20]. Higher-velocity players are at a higher risk for injury, which could be contributing to the increasing rate. Another notable trend is the decreasing incidence of head injuries in the NHL over the studied seasons. In 2019-2020, a rule was implemented that required players who lose their helmets to retrieve their helmet or leave the ice [30]. Since then, there seems to be a downward trend of head injuries in the NHL.

Limitations

The study was retrospective, and as with any study that relies on publicly available data, there is a risk for misreported data. The authors did not have access to the players’ electronic medical records, so the paper is limited in the detail of the reported injuries. All attempts were made to obtain relevant information from the injured players. However, some injury information, such as those injuries that are “undisclosed,” remains withheld by the players. Substantial efforts were made to account for differences in rule changes, game schedules, and roster sizes. However, there may exist other confounding factors that we were not able to account for in the analysis.

## Conclusions

While the NHL adapted their operations during the COVID-19 pandemic in order to minimize the spread of the virus while maximizing games played, this may have contributed to the increase in injury rate during the 2019-2020 playoffs and 2020-2021 season. Changing the offseason training regimens may have led to suboptimal conditioning and preparation for the season. Goalies suffered a disproportionate increase in injuries relative to other positions. There was no observed change in the proportion of injuries relative to certain age groups. The results of the study highlight the need for proper conditioning, sufficient rest in between games, and the importance of the preseason. This ensures a gradual increase in physical load for athletes and ample recovery time, reducing their chance of injury for the rest of the season. Coaches, athletes, and executives should use these findings to guide future changes in schedules and training regimens. Caution should be taken when modifying traditional training schedules. Results from this study can additionally be used to understand the epidemiology of NHL injuries by healthcare professionals and team doctors. Customized treatment and return to play protocols may allow players to maximize their playing time and reduce the likelihood of recurrent injury. Overall, this study provides knowledge of NHL injuries that can be used to prioritize player safety in future decision-making.
